# Fe-N Co-Doped Titanium Dioxide Nanoparticles Induce Cell Death in Human Lung Fibroblasts in a p53-Independent Manner

**DOI:** 10.3390/ijms22179627

**Published:** 2021-09-06

**Authors:** Ionela Cristina Nica, Miruna S. Stan, Roua G. Popescu, Nicoleta Nicula, Robert Ducu, Lucian Diamandescu, Anca Dinischiotu

**Affiliations:** 1 Department of Biochemistry and Molecular Biology, Faculty of Biology, University of Bucharest, 91-95 Splaiul Independentei, 050095 Bucharest, Romania; cristina.nica@drd.unibuc.ro (I.C.N.); roua.popescu@drd.unibuc.ro (R.G.P.); anca.dinischiotu@bio.unibuc.ro (A.D.); 2 Research Institute of the University of Bucharest–ICUB, University of Bucharest, 050657 Bucharest, Romania; 3 Environment/Energy and Climate Change Department, National Institute for Research and Development in Electrical Engineering ICPE—CA, 313 Splaiul Unirii, 030138 Bucharest, Romania; nicoleta.nicula@icpe-ca.ro (N.N.); robert.ducu@icpe-ca.ro (R.D.); 4 National Institute of Materials Physics (NIMP), Atomistilor 405A, Magurele, 077125 Bucharest, Romania; diamand@infim.ro

**Keywords:** TiO_2_, lung fibroblasts, autophagy, apoptosis, cathepsin, lysosome membrane permeabilization

## Abstract

The advancement of nanotechnology in the last decade has developed an abundance of novel and intriguing TiO_2_-based nanomaterials that are widely used in many sectors, including industry (as a food additive and colorant in cosmetics, paints, plastics, and toothpaste) and biomedicine (photoelectrochemical biosensing, implant coatings, drug delivery, and new emerging antimicrobial agents). Therefore, the increased use of engineered nanomaterials in the industry has raised serious concern about human exposure and their unexpected cytotoxic effects. Since inhalation is considered the most relevant way of absorbing nanomaterials, different cell death mechanisms induced in MRC-5 lung fibroblasts, following the exposure to functionalized TiO_2_ NPs, were investigated. Long-term exposure to TiO_2_ nanoparticles co-doped with 1% of iron and nitrogen led to the alteration of p53 protein activity and the gene expression controlled by this suppressor (NF-kB and mdm2), DNA damage, cell cycle disruptions at the G2/M and S phases, and lysosomal membrane permeabilization and the subsequent release of cathepsin B, triggering the intrinsic pathway of apoptosis in a Bax- and p53-independent manner. Our results are of major significance, contributing to the understanding of the mechanisms underlying the interaction of these nanoparticles with in vitro biological systems, and also providing useful information for the development of new photocatalytic nanoparticles that are active in the visible spectrum, but with increased biocompatibility.

## 1. Introduction

Nanosized titanium dioxide (TiO_2_) has high mechanical strength and good corrosion resistance [1], as well as photocatalytic activity [2]. In addition to its highly oxidative, antibacterial [3] and self-cleaning [4] properties, TiO_2_ also has excellent photostability, being the most available and affordable photocatalyst known to date [5]. Unfortunately, the photocatalytic activity of TiO_2_-based materials and their practical applications have two major limitations: the fast recombination of electron–hole pairs, and the light absorption capacity that is only in the ultraviolet (UV) spectrum because of the high energy value of the TiO_2_ band gap [6]. For this reason, continuous efforts to improve the photoactivity of TiO_2_ have been made, and a series of modified materials with increased photocatalytic efficiency have been developed [7,8]. However, the properties that make these nanomaterials highly fascinating and useful for a modern lifestyle raise serious concerns regarding human health and safety issues.

According to the Food and Drug Administration of the United States (USFDA), when it is used as an additive, “TiO_2_ should not exceed 1% of the total weight of the product” [9]. For example, in the commercial sunscreens sold in the market, the concentration of TiO_2_ NPs ranges between 3% and 15%. Therefore, a person applying 5 mL of lotion or cream is exposed to ~150–750 mg NPs (depending on the concentration of TiO_2_ in the cream) [10]. At the European level, according to the latest regulations that were put into place in 2008 and 2012, TiO_2_ is authorized as a food additive in both anatase and rutile form [11,12], although the European Food Safety Authority (EFSA) has, so far, failed to set a recommended daily dose, due to the lack of an extended 90-day toxicity study [13]. Recently, the Risk Assessment Committee of the European Chemicals Agency (ECHA) declared TiO_2_ as suspected of causing cancer through the inhalation route (category 2), due to a lack of sufficient evidence to classify it in the more severe category for carcinogenicity (category 1B).

Toxicity induced by TiO_2_ nanoparticles (NPs) is mostly determined by their physicochemical properties (surface area, crystal form, zeta potential or aggregate size) [14]. It is already known that, due to their surface properties and their interactions with cellular components, TiO_2_ NPs can generate oxidative stress and, consequently, cause cytotoxicity in lung [15,16], hepatic [17], renal [18] and cardiac [19] cells, and also in the central nervous system [20] and circulatory system [21]. More and more studies regarding the cytotoxicity of unmodified metal oxide NPs (TiO_2_, CeO_2_, CuO, ZnO) reported that reactive oxygen species (ROS) accumulation, DNA oxidative lesions and mitotic defects lead to the activation of the intrinsic [22,23,24] and extrinsic pathways of apoptosis [25,26], as well as autophagy [27] and other alternative programmed cell death pathways (such as necroptosis, pyroptosis, paraptosis, mitochondrial permeability transition (MPT)-driven necrosis, parthanatos, pyronecrosis and mitotic or lysosomal cell death [28]).

Taking into consideration the exacerbated synthesis of functionalized NPs, whose effects on human cells have not been thoroughly investigated, and the fact that inhalation is considered to be the most relevant way of absorbing nanomaterials, the present study aims to characterize, using in vitro studies, the cell death mechanisms activated in MRC-5 lung fibroblasts following the exposure to TiO_2_ NPs that are co-doped with 1% atoms of iron and nitrogen (TiO_2_–Fe(1%)-N) synthesized under hydrothermal conditions, using a pH value of ~8.5. The Fe-N doping was performed in order to increase the photoactivity of TiO_2_ NPs in the visible light region. The synergistic effect of these two elements was previously explained by the ability of nitrogen to cause a band-gap narrowing that allows the absorption of longer wavelengths from the visible light spectrum, while iron increases the efficiency of charge separation [29]. In a previous work (focused on the evaluation of the effects triggered, in vitro, in lung and dermal cells, by TiO_2_ NPs that were synthesized under different pH values) we already showed that NPs of this type exerted a more pronounced cytotoxic effect than those that were obtained under acidic conditions (pH~5.5) in the same cell line [29]. Therefore, to investigate the cytotoxicity of these nanoparticles in a more comprehensive way, the expressions of p53, NF-kB, MDM-2, Bax, Bcl-2, LC3 and caspase-3 proteins, as well as the fibroblasts’ distribution over the cell cycle, were analyzed using Western blot, Real Time PCR (RT-PCR) and flow cytometry in the present study. At the same time, information regarding the interaction between synthesized TiO_2_ NPs and DNA, as well as on genetic damage level, was obtained using Comet assay and an 8-hydroxy 2-deoxyguanosine (8-OHdG) test.

## 2. Results

### 2.1. Physicochemical Characterization of TiO_2_ NPs

**Figure 1:**
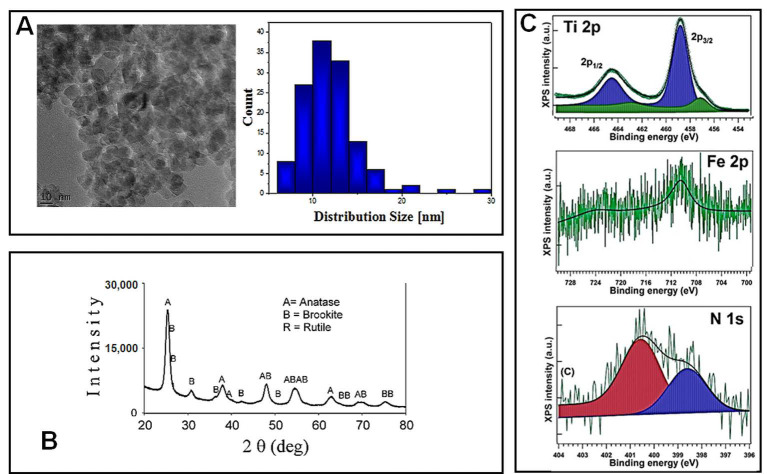
(**A**) Transmission electron microscopy (TEM) images and particle distribution diagrams; (**B**) X-ray diffraction (XRD) patterns and phase assignment; (**C**) The X-ray photoelectron spectroscopy (XPS) spectra of the TiO_2_–Fe(1%)-N (pH~8.5) sample.

Before being tested for their cytotoxicity and genotoxicity effects on lung cells, TiO_2_–Fe(1%)-N (pH~8.5) NPs were characterized using electron microscopy and spectroscopy techniques. Transmission electron microscopy (TEM) was used in order to reveal the morphology of nano-scaled photocatalysts powders, as well as the corresponding particle distribution (Figure 1A). The TiO_2_–Fe (1%)-N (pH~8.5) particles showed a mean size of 10 ± 4 nm, with these results being in good agreement with the XRD profile. Additionally, TEM and SEM investigations displayed a prevailing quadratic morphology of the TiO_2_ NPs obtained at pH~8.5.

The X-ray diffractograms presented in Figure 1B, and the corresponding Rietveld refinements results reported in Table 1, show that the analyzed TiO_2_–Fe(1%)-N (pH~8.5) samples consisted in nano-scaled anatase (~85%) and brookite (~15%).

The presence of iron and nitrogen atoms in the hydrothermally prepared nanoparticles was revealed through the XPS measurements. Previously obtained XPS spectra of Ti 2p, Fe 2p and N 1s, with their corresponding binding energies, are presented in Figure 1C and Table 2, respectively. Ti 2p spectra exhibited a peak at 458.08–458.88 eV, representing a pattern of Ti^4+^ state in TiO_2_. The 460.27 eV peak could be associated with a shake-up satellite, and the 456.88 eV peak indicates the presence of Ti^3+^ ions [29], likely through the formation of a small amount of a TiFeO_3_-like compound, considering the Fe^3+^ chemical state of iron. A more detailed analysis of these hydrothermally synthesized TiO_2_ NPs was previously presented [29].

Additionally, in our previous work, we already reported data regarding the Z-average, polydispersity index (PdI) and zeta potential values that were measured in a cell culture medium for up to 72 h [29]. We considered that the zeta potential values revealed the most realistic overview regarding the suspensions’ stability and behavior over time, showing that just before the cell culture exposure, co-doped TiO_2_ NPs suspensions were near the limit of stability (~30 mV). However, after the first 24 h, the zeta potential value diminished, and it was further maintained until the end of the incubation period (72 h). Additionally, as shown in the phase contrast microscopy images, these nanoparticles tend to aggregate, and to precipitate on the surface of cells.

### 2.2. Influence of TiO_2_ NPs on Cell Viability of MRC-5 Fibroblasts

Three intervals of exposure (24, 48 and 72 h) and an increasing dose range (between 0.001 and 25 μg/mL) were selected for the TiO_2_–Fe(1%)-N pH~8.5 NPs. In this study, we attempted to capture the dynamics of time modulation for the proteins involved in different cell death pathways. However, when testing several high doses of TiO_2_ NPs (greater than 100 μg/mL), we noticed a total disappearing of the main proteins of interest (NF-kB and p53) in the exposed cells. Therefore, this dynamic could only be analyzed for doses between 0.001 and 25 μg/mL.

**Figure 2:**
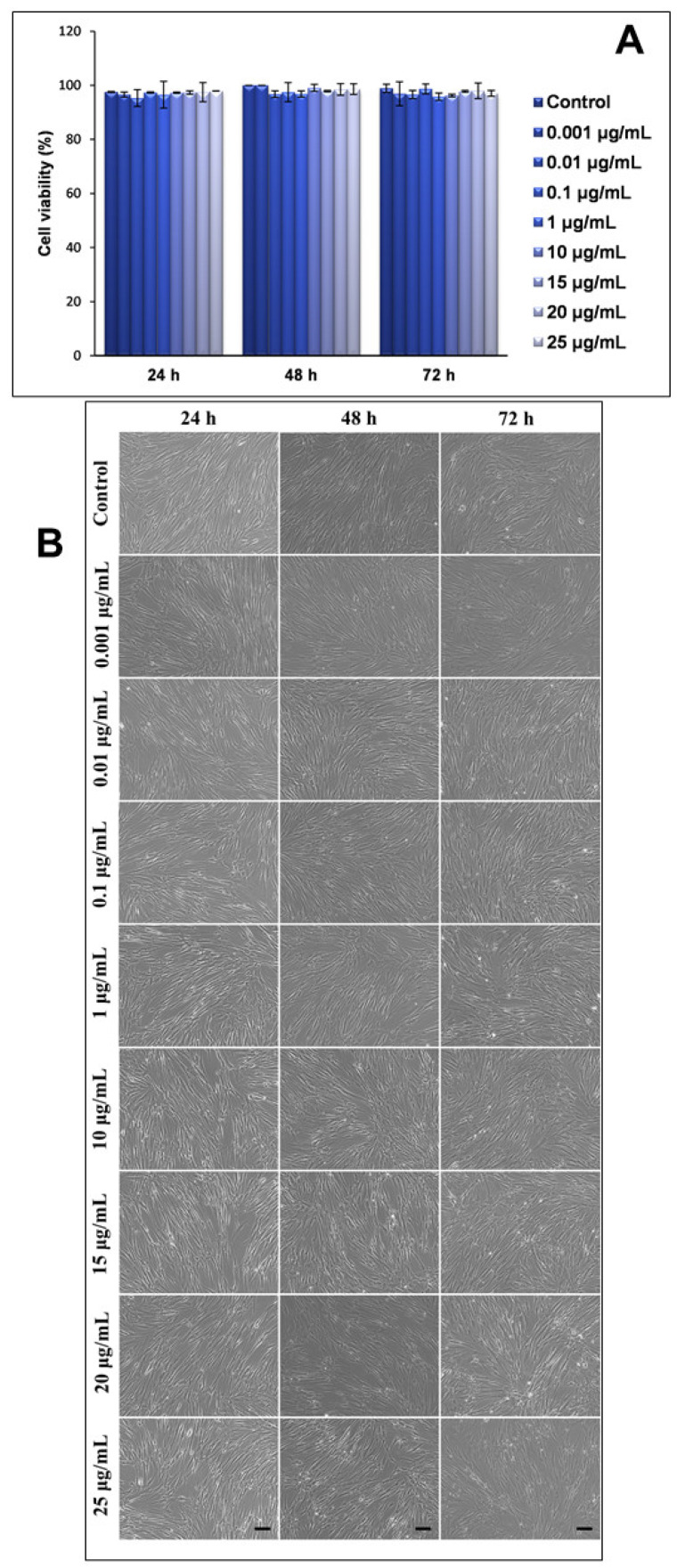
Cell viability and morphology of MRC-5 lung fibroblasts after exposure to different concentrations (μg/mL) of TiO_2_–Fe(1%)-N (pH~8.5) NPs for 24, 48 and 72 h, as shown by Trypan blue exclusion test (**A**) and phase contrast microscopy (**B**). Results are expressed as the mean ± standard deviation (SD) (*n* = 3) and represented relative to the untreated cells (control). Scale bar: 50 μM.

Firstly, the influence of NPs of this type on the viability of human lung fibroblasts (MRC-5 line) was assessed using a Trypan blue exclusion test. Our results showed that neither the concentration of NPs in the culture medium, or the exposure intervals, significantly influenced the viability of the lung cells, with all values being very close to those that were recorded for the untreated cells (control) (Figure 2A). These results were also correlated by the microscopy images that were captured at the same time intervals and concentrations of NPs (Figure 2B).

Secondly, the morphological changes of the MRC-5 cells that were exposed to increasing concentrations of TiO_2_–Fe(1%)-N NPs, with improved photocatalytic properties, were analyzed using phase contrast microscopy, and the obtained results were consistent with the viability test shown in Figure 2A. Thus, human lung fibroblasts maintained their specific morphology after 24, 48 and 72 h (with no significant differences between the samples and the control), and their proliferative capacity was not significantly impaired in the presence of nano-photocatalysts.

### 2.3. Influence of TiO_2_ NPs on Proteins Involved in Apoptosis of Lung Fibroblasts

To evaluate the ability of TiO_2_ NPs to induce apoptosis, the protein expression levels of p53, NF-kB, MDM2, Bcl-2 and Bax, as well as pro-caspase and caspase 3, were quantified using Western blot analysis.

The p53 protein plays a key role in activating the cell cycle checkpoints in the G1 and G2/M phases, DNA repair and programmed cell death pathways, in order to maintain genomic stability [30]. Under normal physiological conditions, p53 is maintained in EK cells in low concentrations, due to proteasomal degradation mediated by MDM2 E3 ubiquitin ligase [31]. When stress signals are detected, p53 can induce either cell cycle arrest or cell death.

**Figure 3:**
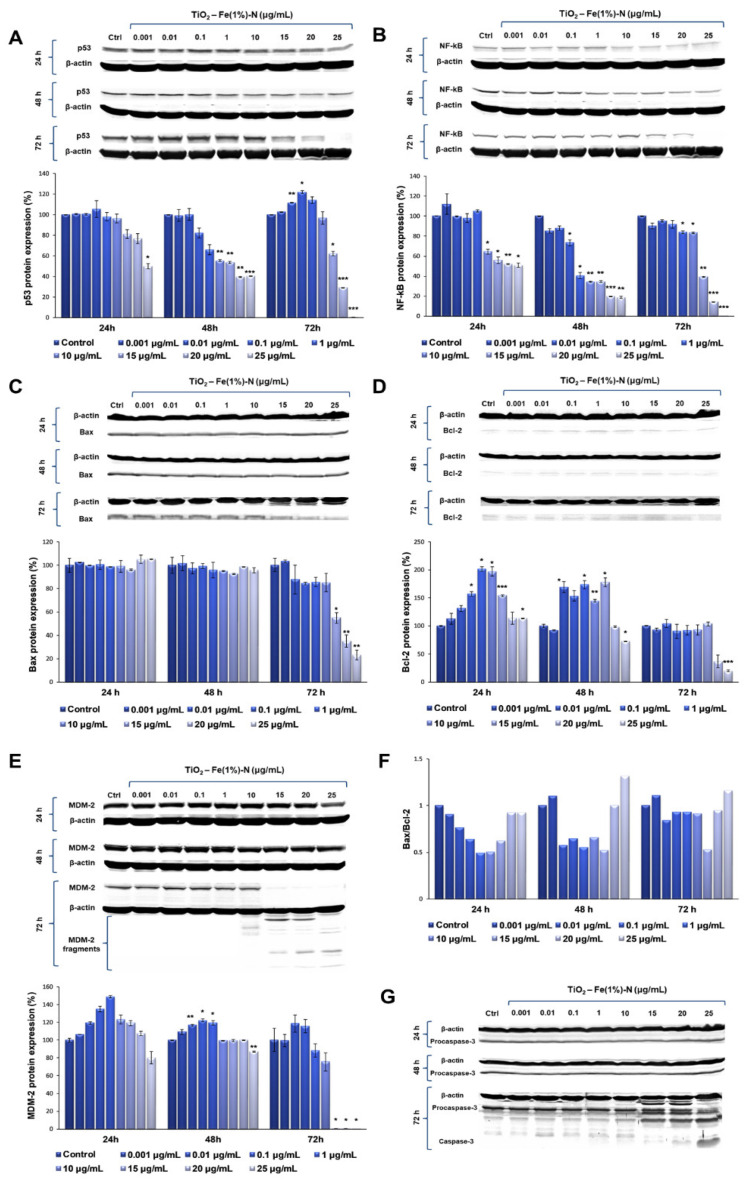
Representative Western blot images and quantitative analyses of p53 (**A**), NF-kB (**B**), Bax (**C**), Bcl-2 (**D**), MDM-2 (**E**), procaspase-3 and caspase-3 protein expression (**G**), as well as Bax/Bcl-2 expression ratio (**F**) in MRC-5 lung fibroblasts after exposure to different concentrations (μg/mL) of TiO_2_–Fe(1%)-N (pH~8.5) NPs for 24, 48 and 72 h. The protein expression was normalized to β-actin. Results are expressed as the mean ± standard deviation (SD) (*n* = 3) and represented relative to the untreated cells (control). * *p* < 0.05, ** *p* < 0.01 and *** *p* < 0.001 compared to control.

Our results showed that TiO_2_ NPs induced significant time- and dose-dependent changes in p53 protein expression (Figure 3A). Thus, after the first 24 h of exposure, p53 protein expression did not change at lower doses of TiO_2_ NPs (0.001 and 0.01 μg/mL); in contrast, it slightly increased in the cells that were exposed to 0.1 μg/mL of TiO_2_ NPs, followed by a gradual diminution of protein expression up to 50%, compared to the untreated cells, recorded to the maximum dose of 25 μg/mL of TiO_2_ NPs. After 48 h, the protein expression of p53 showed a similar trend, which means that there were no changes compared to the control cells for the lower doses, although, this time, both doses of 20 and 25 μg/mL of TiO_2_ NPs induced the same decrease in p53 protein expression by ~60% of the control value. However, the most prominent changes were observed after 72 h of incubation, when the expression level of p53 protein recorded a maximum increase of ~20%, compared to the control, at a 0.1 μg/mL TiO_2_ NP dose, and was completely inhibited in the presence of 25 μg/mL of TiO_2_ NPs.

Besides p53, the nuclear factors NF-kB are the main molecules that influence cell survival and division in response to their exposure to cytotoxic or genotoxic agents [32]. Protein p53 can induce NF-kB activation, and the inactivation of NF-kB cancels the p53-mediated apoptotic response, without affecting the ability of this protein to modulate target gene expression, or block cells in one phase of the cell cycle [33]. Even though several studies indicated that, following DNA damage in malignantly transformed cells, p53 and NF-kB can interact through a variety of mechanisms, a research group led by Gerondakis failed to establish functional links between NF-kB and p53 pathways [34]. NF-kB did not influence the p53-dependent apoptosis and cell cycle arrest. These findings could lead to the hypothesis that p53, or the NF-kB-mediated response, and the relationship between them, differ from one cell type to another.

Following the evaluation of NF-kB expression after the exposure of pulmonary fibroblasts to different concentrations of TiO_2_–Fe(1%)-N NPs, a similar pattern was observed (Figure 3B). After the first 24 h of incubation with 0.001 μg/mL TiO_2_ NPs, there was a 12% increase in NF-kB protein expression, compared to the control. Additionally, its level recorded a sharp decrease of almost 50% in the lung cells that were exposed to 15 μg/mL TiO_2_ NPs, in comparison to the control, which was maintained up to the highest dose of 25 μg/mL. After 48 h of incubating the pulmonary fibroblasts in the presence of TiO_2_ NPs, a dose-dependent decrease in NF-kB expression was observed, culminating in a relative expression of NF-kB protein of only 18% in the cells that were exposed to 20 and 25 μg/mL of TiO_2_ NPs. Similarly to the modulation of p53 expression, the exposure of the pulmonary fibroblasts to the modified TiO_2_ NPs for 72 h led to the most significant variations in NF-kB protein expression. Thus, for lower concentrations (0.001–10 μg/mL) of NPs, a slight decrease was recorded (up to 18%, compared to the control) in a dose-dependent manner, while the exposure of lung cells to 15 μg/mL TiO_2_ NPs resulted in a significant decrease of 60% in the NF-kB level, compared to the control value. Moreover, the highest concentration of 25 μg/mL induced a total lack of this transcription factor.

Since MDM-2 is the major regulator of p53 transcription, the influence of TiO_2_ NPs on MDM-2 protein expression was also investigated in this study. Our data showed that the 24 h exposure of lung fibroblasts to concentrations between 0.001 and 1 μg/mL of TiO_2_–Fe(1%)-N NPs resulted in a dose-dependent increase, with a maximum value of relative protein expression of 148%. Subsequently, a 20% decrease in the MDM-2 level was observed for the highest dose of 25 μg/mL TiO_2_ NPs. The same trend was observed after 48 h of incubation, although the maximum value of MDM-2 expression was recorded at 0.1 μg/mL NPs, and the increase was only 22%, compared to the control. In addition, the presence of 25 μg/mL TiO_2_ NPs in culture media resulted in a 13% decrease in protein expression, compared to the untreated cells. The influence of TiO_2_ NPs on MDM-2 protein expression in the lung cells had a more pronounced effect after 72 h, when the protein expression slightly increased by ~15%, compared to the control, when exposed to 0.01 and 0.1 μg/mL TiO_2_ NPs. Afterwards, the MDM-2 level began to decrease, and all doses of TiO_2_ NPs that were higher than 15 μg/mL determined the cleavage of MDM-2 and, thus, most likely, its inactivation (Figure 3E).

Bax and Bcl-2 are two other important proteins involved in the regulation of apoptosis, which can form heterodimers, and the overexpression of one antagonizes the effect of the other. They belong to two classes of proteins in the Bcl-2 family: one class includes Bcl-2 and Bcl-XL, which can inhibit apoptosis, and the other class includes proteins such as Bax, Bak and Bcl-XS, which promote apoptosis [35]. It is well known that the Bax/Bcl-2 expression ratio determines the susceptibility of cells to apoptosis. Thus, a Bax/Bcl-2 ratio of <1 is characteristic for resistant cells, while a Bax/Bcl-2 ratio of >1 promotes the activation of caspase-3 and the onset of apoptosis [36,37].

The results obtained in this study showed that both pro-apoptotic factor Bax, and anti-apoptotic Bcl-2, followed the same trend observed for the other proteins analyzed, with a significant decrease in protein expression following the exposure to 25 μg/mL of TiO_2_–Fe(1%)-N NPs for 72 h. Regarding the Bax/Bcl-2 expression ratio, a very slight increase was observed in the case of 0.001 and 25 μg/mL doses after 48 and 72 h of exposure, although it is too insignificant to be able to relate it to the initiation of apoptosis through the Bax-dependent intrinsic mitochondrial pathway.

Finally, to evaluate the ability of TiO_2_–Fe(1%)-N (pH~8.5) NPs to induce the apoptosis of lung cells, we evaluated the expression of procaspase-3 and the activation of caspase-3—the main apoptotic effector. Our results showed that MRC-5 fibroblasts that were exposed to doses of TiO_2_ NPs ranging between 0.001 and 10 μg/mL for 72 h maintained the level of procaspase-3 in the cytosol that was close to the control (Figure 3G). In contrast, it was observed that the incubation of lung cells with TiO_2_ NP doses that were greater than or equal to 15 μg/mL resulted in a progressive decrease in inactive precursor expression that was proportional to the accumulation of cleaved caspase-3 (Table 3). These data indicate that TiO_2_ NPs with photocatalytic activity in the visible spectrum induced proteolytic cleavage and caspase-3 activation at high doses (above 25 μg/mL), thus triggering apoptosis, albeit in a Bax- and p53-independent manner.

### 2.4. Dose-Dependent Autophagic Effect of TiO_2_ NPs on Lung Fibroblasts

**Figure 4:**
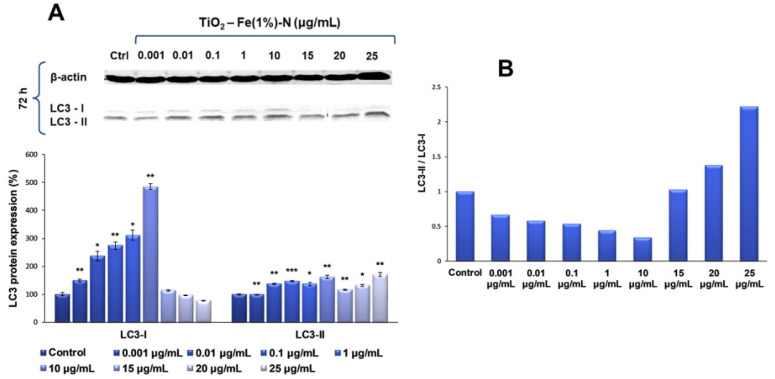
Representative Western blot images and quantitative analyses of LC3-I and LC3-II protein forms expression (**A**), and LC3-II/LC3-I expression ratio (**B**) in MRC-5 lung fibroblasts after exposure to different concentrations (μg/mL) of TiO_2_–Fe(1%)-N (pH~8.5) NPs for 72 h. The expression of target proteins was normalized to that of β-actin. Results are expressed as the mean ± standard deviation (SD) (*n* = 3) and represented relative to the untreated cells (control). * *p* < 0.05, ** *p* < 0.01 and *** *p* < 0.001 compared to control.

Since the previous analyses showed that the most significant changes occurred in TiO_2_ NPs-treated cells after 72 h of exposure, the influence of TiO_2_–Fe(1%)-N (pH~8.5) NPs on the expression of LC3, a protein with a key role in the formation and maturation of autophagosome, was further analyzed, specifically for this interval. In this way, our results showed that the presence of TiO_2_ NP doses ranging between 0.001 and 10 μg/mL in the culture media for 72 h led to a progressive, dose-dependent increase in the LC3-I expression of lung fibroblasts of up to five times the amount in the control, followed by a level diminution of ~25% of the control, for the highest dose of 25 μg/mL (Figure 4). In contrast, the level of LC3-II protein form was maintained at close to the control value, with only a slight increase of approximately 40% and 60%, respectively, for 0.01 and 10 μg/mL TiO_2_ NP doses. It is noteworthy that, at higher doses (15, 20 and 25 μg/mL) of photocatalytic NPs, the LC3-I level decreased, and the LC3-II expression began to increase, suggesting that these doses promoted the conversion of LC3-I to its phosphatidylethanolamine-conjugated form (LC3-II), which is incorporated into the autophagosome membrane, initiating the autophagy process.

### 2.5. Gene Expression Modulation of TiO_2_ NPs in Lung Fibroblasts

**Figure 5:**
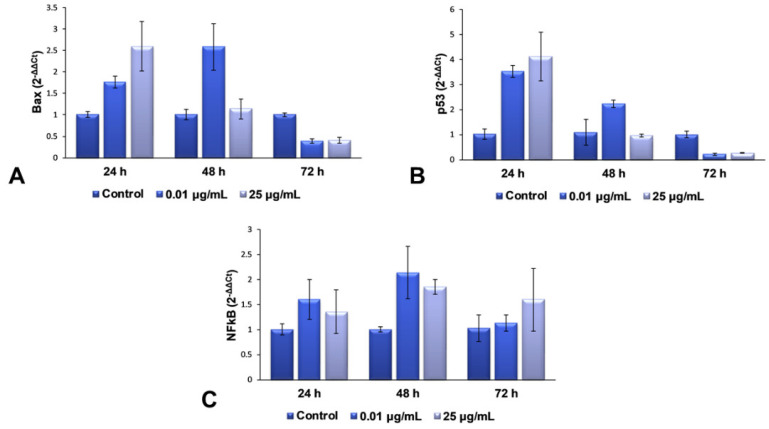
RT-PCR analysis of the expression changes in mRNA levels of Bax (**A**), p53 (**B**) and NFkB (**C**) from MRC-5 cells after exposure to different concentrations (μg/mL) of TiO_2_–Fe(1%)-N (pH~8.5) NPs for 24, 48 and 72 h. Results are expressed as the mean ± standard deviation (SD) (*n* = 3), normalized by subtracting the arithmetic mean of 18S RNA and gapdh reference genes from each gene of interest, and represented relative to the untreated cells (control).

For the second part of the present research, we decided to continue our studies with only the maximum dose (25 μg/mL) and an intermediary one (0.01 μg/mL), in order to elucidate the complete mechanism of toxicity that is activated in lung cells exposed to more than 25 μg/mL of TiO_2_–Fe(1%)-N (pH~8.5) NPs. The mRNA levels of Bax, p53 and NFkB genes were quantified using RT-PCR, to evaluate the effect of the interaction between NPs and cells on the genes in the structure of chromosomes. These three genes were selected for qPCR, to verify whether gene expression coincides with the protein expression determined by Western blot analysis. The results obtained and presented in Figure 5 highlight a dose-dependent induction of the transcription of Bax and p53 genes after 24 h of incubation with TiO_2_ NPs, compared to the control cells, followed by their down-regulation after 72 h for both of the tested concentrations. This change correlated very well with the results observed in the Comet test, as well as with the increased levels of 8-OHdG after 24 h.

All of these results suggest that the TiO_2_ NPs induce a change in the structure of the genetic material, resulting in the hydroxylation of deoxyguanosine, and the initiation of DNA repair mechanisms. The accumulation of defects due to prolonged exposure to NPs (72 h) causes a complete change in the DNA structure of fibroblasts’ chromosomes, almost completely inhibiting the expression of genes that could block the cell cycle and initiate the death of defective cells. In the case of NFkB gene expression, an up-regulation trend towards the control was observed, suggesting an activated proinflammatory effect, due to cell exposure to TiO_2_ NPs.

### 2.6. Influence of TiO_2_ NPs on MRC-5 Fibroblasts’ Distribution over the Cell Cycle

**Figure 6:**
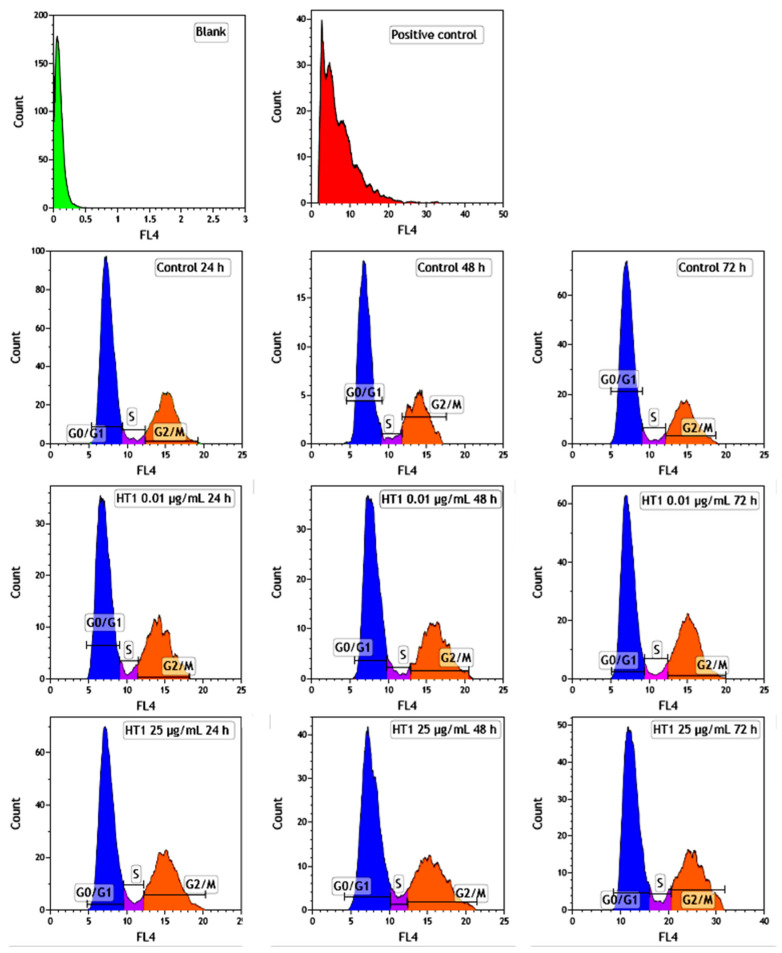
Analysis of cell cycle progression in MRC-5 lung fibroblasts exposed to different concentrations (μg/mL) of TiO_2_–Fe(1%)-N (pH~8.5) NPs for 24, 48 and 72 h. The compound used as the positive control was dimethyl sulfoxide (25 µg/mL). Results are expressed as the mean ± standard deviation (SD) (*n* = 3).

**Figure 7:**
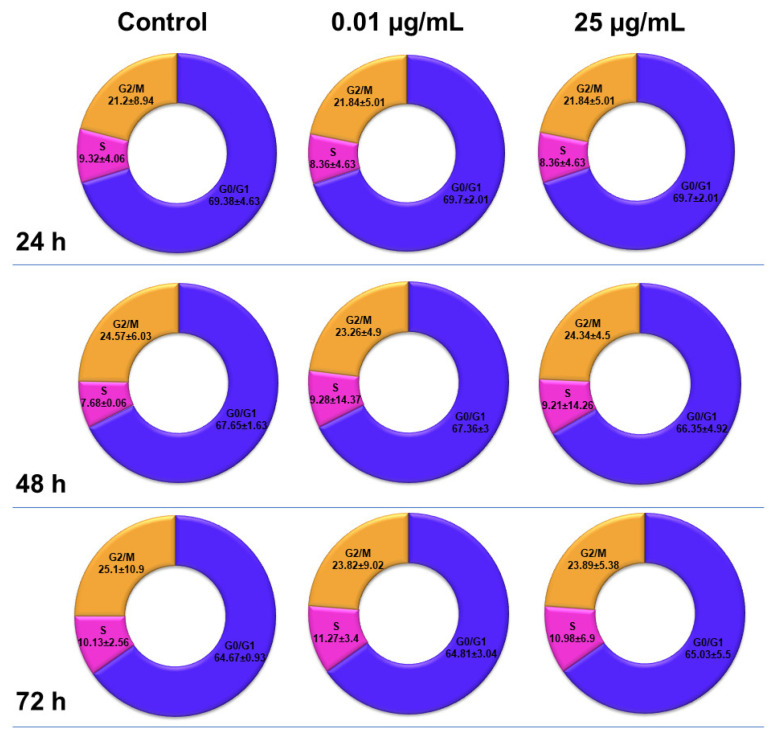
MRC-5 lung fibroblasts distribution over the cell cycle after exposure to different concentrations (μg/mL) of TiO_2_–Fe(1%)-N (pH~8.5) NPs for 24, 48 and 72 h. Results are expressed as the mean ± standard deviation (SD) (*n* = 3).

The distribution of MRC-5 pulmonary fibroblasts over the cell cycle phases, following exposure to TiO_2_ photocatalytic NPs, was analyzed using flow cytometry, and the results obtained after data acquisition and quantification are shown in Figure 6 and Figure 7.

Flow cytometry analysis showed that, after the first 24 h of exposure to co-doped TiO_2_ NPs, the number of lung fibroblasts in the G0/G1 phase began to decrease, while an increasing number of cells were found in the S and G2/M phases. After 48 h of incubation, this pattern of cell distribution was maintained, with an increase in the number of cells in the S and G2/M phases in a dose-dependent manner, although the maximum dose of 25 μg/mL of TiO_2_ NPs caused a similar increase in the number of cells in both the S and G2/M phases. In contrast, after 72 h, it was observed that the distribution of cells in the S phase was higher than the G2/M phase, after exposure to 0.01 μg/mL TiO_2_ NPs. Moreover, the incubation of MRC-5 cells in the presence of 25 μg/mL TiO_2_ NPs resulted in an S phase increase of almost 25% over the control, while the G2/M phase registered an elevation of only 9% over the control.

### 2.7. DNA Strand Breaks and Oxidation Induced by TiO_2_ NPs

In the present study, the degree of nuclear DNA damage induced by TiO_2_ NPs was evaluated and quantified using a Comet assay performed in alkaline conditions. Unlike the classical method with Tris-borate-EDTA buffer (TBE), electrophoresis in alkaline medium extends the detection range of strand breaks, without changing the sensitivity of the method represented by the minimum fragmentation threshold that can be detected, and also ensures a more pronounced comet tail profile [38].

**Figure 8:**
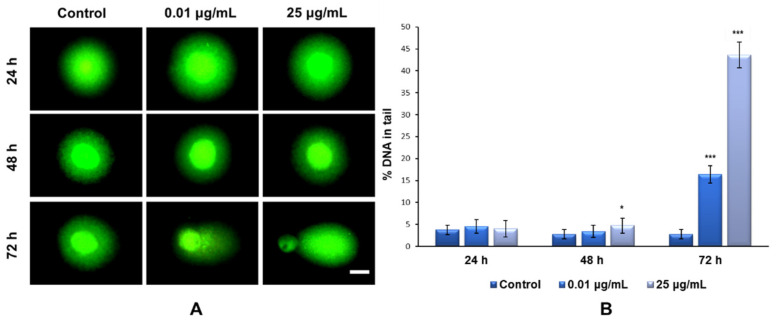
Representative fluorescence images of MRC-5 cell morphology (**A**) and their genotoxic response (**B**) after exposure to different concentrations (μg/mL) of TiO_2_–Fe(1%)-N (pH~8.5) NPs for 24, 48 and 72 h. Results are expressed as the mean ± standard deviation (SD) (*n* = 3) and represented relative to the untreated cells (control). * *p* < 0.05 and *** *p* < 0.001 compared to control. Scale bar: 50 μM. Magnification: ×100. The green fluorescence intensity was analyzed and quantified using the OpenComet program.

Our results showed that the exposure of MRC-5 fibroblasts to the culture media containing 0.01 and 25 μg/mL of TiO_2_–Fe(1%)-N (pH~8.5) NPs for 24 and 48 h did not induce the formation of DNA strand breaks (Figure 8A). In contrast, after 72 h of incubation with the same doses of NPs, there was a significant increase in the percentage of nuclear DNA in the comet tail; up to 16% for the 0.01 μg/mL dose and 43% for the maximum dose of 25 μg/mL, compared to the 3% recorded for the control (Figure 8B).

**Figure 9:**
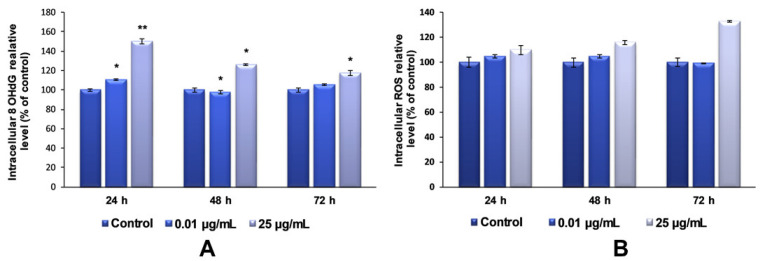
The 8-OHdG (**A**) and ROS (**B**) level measured in MRC-5 cells after exposure to different concentrations (μg/mL) of TiO_2_–Fe(1%)-N (pH~8.5) NPs for 24, 48 and 72 h. Results are expressed as the mean ± standard deviation (SD) (*n* = 3) and represented relative to the untreated cells (control). * *p* < 0.05 and ** *p* < 0.01 compared to control.

Regarding the investigation of TiO_2_–Fe(1%)-N NP-induced oxidative DNA-damage, our data showed that the maximum dose of photocatalytic NPs (25 µg/mL) induced an increase in the 8-OHdG level after each tested interval, compared to the control (Figure 9A). It was also observed that this increase was inversely proportional to the exposure time. This result can be attributed to the strong aggregation behavior of TiO_2_ NPs, which is very well known. According to the Derjaguin–Landau–Verwey–Overbeek (DLVO) theory of colloidal stability, TiO_2_ NPs have the greatest tendency to form large aggregates [39].

The induction of cytotoxicity and oxidative damage to biomolecules is due to ROS generation that produces oxidative stress. Therefore, to assess whether TiO_2_–Fe(1%)-N (pH~8.5) NPs induced oxidative stress, the intracellular ROS level was measured using the fluorescence intensity of 2′,7′-dichlorodihydrofluorescein diacetate (DCFDA). The exposure of MRC-5 lung fibroblasts to 0.01 μg/mL of TiO_2_ NPs for 24 and 48 h resulted in an insignificant increase in the ROS level, by only 4% relative to the control. In contrast, the cells that were incubated with culture media containing 25 ug/mL showed a time-dependent elevation in intracellular ROS level, with a maximum increase of 32%, compared to the untreated cells, recorded after 72 h.

### 2.8. Lysosomal Modifications Induced by TiO_2_ NPs in Lung Fibroblasts

**Figure 10:**
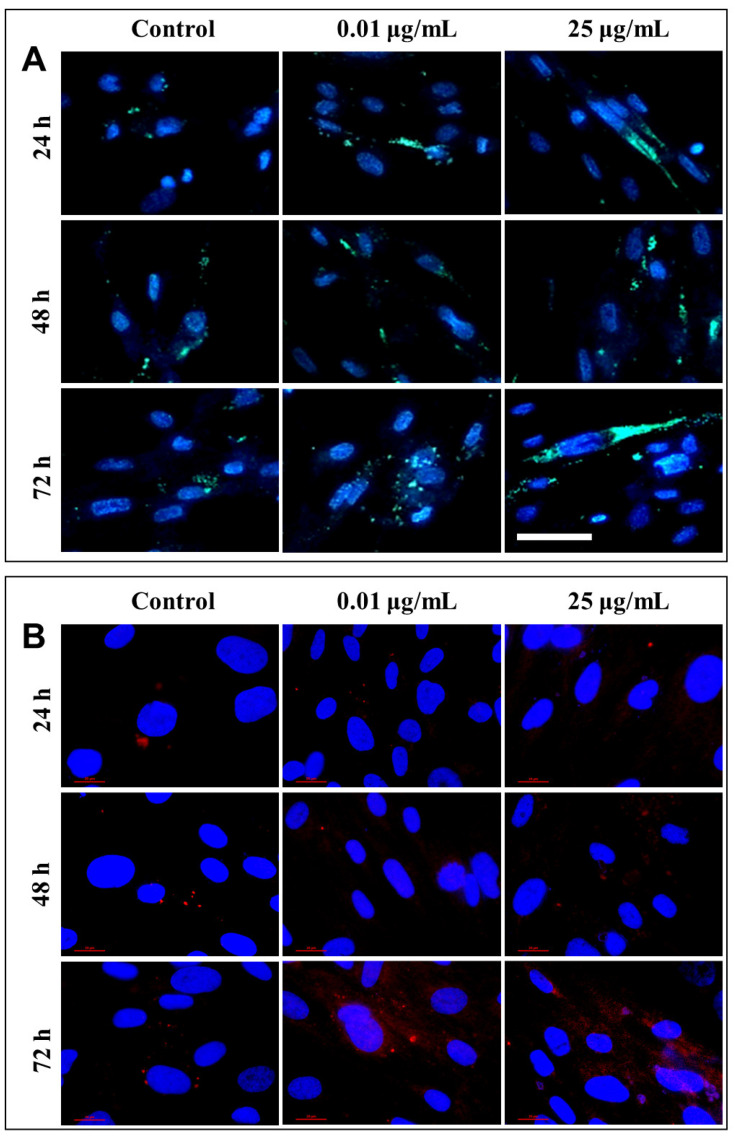
Representative images of fluorescence microscopy showing the lysosomal changes induced in MRC-5 cells after exposure to different concentrations (μg/mL) of TiO_2_–Fe(1%)-N (pH~8.5) NPs for 24, 48 and 72 h. (**A**) intracellular distribution of lysosomes (stained in green) in human lung fibroblasts (nuclei labeled in blue). Scale bar in the lower left corner is the same for all images: 50 μm. (**B**) the lysosomal membrane permeabilization and subsequent release of cathepsin B (stained in red) into the cytosol (nuclei labeled in blue). Scale bar in the lower left corner: 20 μm.

After each exposure, a similar distribution of lysosomes was observed in the lung cells, with no significant differences between the untreated cells and those that were exposed to different concentrations of TiO_2_ NPs. Only a slight increase in lysosomal density was recorded in fibroblasts incubated for 72 h with 25 µg/mL of photocatalytic NPs (Figure 10A). Therefore, in order to confirm cell death by lysosomal degradation, additional studies were performed to highlight the lysosomal membrane permeabilization (LMP), and the consequent release of cathepsins in the cytosol.

Under normal physiological conditions, in healthy cells, cathepsin is localized in lysosomes as highly fluorescent red vesicles, while the LMP causes their release and results in a diffused staining pattern throughout the entire cytosol. Fluorescent labeling through the immunocytochemistry of cathepsin B, the most abundant lysosomal protease, provided a visualization of its release from the lysosomal lumen to the cytosol, following the permeabilization of the lysosomal membrane (Figure 10B). This phenomenon was observed in cells that were exposed to 0.01 and 25 µg/mL TiO_2_–Fe(1%)-N (pH~8.5) NPs for 72 h.

## 3. Discussion

Eukaryotic cells possess a series of regulators that control the entry and proper progression through the cell cycle. When stress signals are detected, they have the ability to stop the cell cycle, providing enough time to restore the chromosomes’ integrity; if the DNA damage is too extensive to be repaired, programmed cell death pathways are activated. Numerous forms of cell death can be induced by specific signaling pathways, depending on the physicochemical properties of the NPs, the dose used and the time exposure. Thus, in tissues or cell cultures that are exposed to the same stimulus, several types of cell death can be observed simultaneously [40].

The reason for which we have chosen to focus our studies on NPs of this type was primarily determined by the existence of more pronounced cytotoxic effects, compared to those induced by the NPs obtained under acidic conditions (pH~5.5), as was shown in a previous work [29]. Given that inhalation is considered to be the most important route of absorption for nanomaterials, and NPs may persist in the lungs even a few years after the cessation of exposure, the MRC-5 cell line was selected as the most relevant experimental model for our subsequent studies. Our previous results have also shown that MRC-5 fibroblasts are more sensitive to TiO_2_ NP exposure, compared to CCD-1070Sk skin fibroblasts [29].

With the recent advances regarding in vitro models, not only in vivo studies, but also cellular studies can be relevant for toxicological testing. However, both approaches have their limitations. Commonly used animal models cannot mimic human exposure, due to differences in breathing patterns, while in vitro systems lack both the complexity of the entire organism, although they are important for identifying cellular mechanisms without ethical and economical concerns [41]. The direct delivery of aerosols to air–liquid interface-cultivated cell cultures could be chosen as a more realistic simulation of the inhalation scenario. In spite of that, most in vitro studies of NP toxicology predominantly use submerged cell cultures exposed to particle suspensions, even if this approach does not fully reflect the natural process [42]. Moreover, comparisons between aerosol and suspension exposures revealed that the general nanomaterials’ toxicity output was similar across the different exposure methods used, and the biological effects were dependent on the timing of the dose delivery [43].

Despite the fact that no correlation has yet been established between NP-induced genotoxicity and lung cancer, epidemiological studies and in vivo experiments revealed that long-term inflammation and the presence of oxidative stress in tissues can induce DNA lesions, mutations or deletions in the genes, which may subsequently lead to the development of tumors and cancer [44]. The p53 protein can also block the G2/M transition due to DNA damage. However, if the DNA lesions are too extensive to be repaired, p53 initiates apoptosis through the transcriptional activation of pro-apoptotic proteins from the Bcl-2 family [45]. Therefore, the loss of the ability to activate the p53 protein increases the chances for normal cells to survive and proliferate in unfavorable conditions. The flow cytometry analyses performed in our study showed that the presence of TiO_2_–Fe(1%)-N (pH~8.5) NPs in the culture media over time led to only slight alterations in the cell cycle phases (Figure 7).

Another study that was performed on commercial P25 Degussa TiO_2_ NPs reported that the cell cycle progression of NIH 3T3 cells and human fibroblast HFW cells was disrupted in the G2/M phase, while abnormal chromosome segregation and centrosome amplification were significantly increased, leading to genomic instability after exposure to 10 µg/mL doses [46]. Similar results indicating the ability of TiO_2_ NPs to exert genotoxic effects mediated by oxidative stress in a time- and dose-dependent manner have been reported in both in vitro [47,48] and in vivo [49] studies. Moreover, the presence of TiO_2_ NPs (0.008–100 µg/mL) favors the formation of micronuclei and polyploids as a result of induced oxidative stress in the peripheral blood lymphocytes and human epidermal cells [10,50]. The 8-hydroxy-2-deoxyguanosine (8-OHdG) is a biomarker of NP-induced oxidative stress in exposed cells. The 8-OHdG lesions formed when the ROS generated by the NPs attack guanine from the chromosome-forming DNA structure. In the case of high NP doses, the difference between the first and last exposure interval (24 h and 72 h), illustrated in Figure 9A, could be explained by the fact that ROS are generated from the first hours of exposure, and they then modify the nucleotides in the DNA structure, although the integrity of the chromosome structure is still maintained overall (as shown in Figure 8 by the Comet assay). However, after three days of exposure, the effect of the NPs becomes stronger, destabilizing the entire DNA structure, while the level of 8-OHdG returns to that of the control, most likely because the cells activated the proofreading mechanisms to correct the altered nitrogenous bases and repair the DNA damage over the cell cycle. However, failure to repair these altered nucleotides is a common cause of mutations, some of them contributing to TiO_2_-induced mutagenesis.

Our results showed that, after the exposure of MRC-5 fibroblasts to TiO_2_–Fe(1%)-N (pH~8.5) NPs for 72 h, caspase-3 is activated concomitantly with the decreased protein expression of other pro-apoptotic factors (p53, NF-kB, Bax). These data indicate that, at high doses (above 25 µg/mL), TiO_2_ NPs with photocatalytic activity in the visible spectrum were able to induce proteolytic cleavage and caspase-3 activation, thus triggering the intrinsic mitochondrial pathway of apoptosis, in a Bax- and p53-independent manner. These results, combined with the MDM-2 cleavage at 15, 20 and 25 μg/mL doses, could indicate cell death by lysosomal alteration. Similar results have already been reported, with numerous studies indicating the activation of pro-apoptotic proteins (Puma, Noxa, Fas and others) on a p53-independent and ROS-dependent pathway [51,52], reactive species accumulation being involved in mitochondrial membrane depolarization, caspase-3 activation and cell cycle arrest in the G2/M phase [53,54]. Previous studies have shown that NF-kB activation can be inhibited by ROS, possibly by oxidizing certain cysteine residues in the NF-kB polypeptide chain or IkB inhibitor kinase (IKK), with ROS-modulated NF-kB activity being dependent on cell type and stimulus [55,56].

Although the overexpression of the mdm2 gene is generally associated with p53 inactivation, as with its substrate, we should take into consideration that MDM-2 protein is also subject to ubiquitylation and degradation, with a very short half-life, explaining its cleavage after the incubation with NPs. In fact, even its intrinsic E3 ubiquitin ligase activity leads to its own ubiquitin labeling [57]. Other studies have shown that long-term exposure to concentrations between 10 and 100 μg/mL of TiO_2_ NPs has led, not only to the disruption of p53 activity, but also to the expression of genes that are regulated by this protein (p21 and mdm2) in response to DNA damage, without affecting the viability of human hepatoma HepG2 cells [58].

Autophagy can be one of the main mechanisms of cell survival that ensures the elimination of damaged organs and biomolecules, under the action of oxidative stress induced by NPs [59]. However, if the stress factor is applied for a longer period, dysfunctions occur in various organs, and autophagy serves as a cell death mechanism, promoting the self-destruction of cells [60]. However, the dual role of autophagy in both cell survival and programmed cell death, as well as the relationship between p53 protein activity and LC3 regulation, differs in a cell type dependent manner. It is well known that, under moderate stress conditions, the p53 protein maintains autophagic homeostasis and ensures the low expression of LC3-I, and a value of the LC3-II/LC3-I ratio lower than 1, promoting cell survival [61]. The loss of p53 activity allows for excessive production of the LC3-I protein, leading to cell death. In this context, the results obtained in the present study showed that, for doses of TiO_2_–Fe(1%)-N (pH~8.5) NPs between 0.01 and 10 μg/mL, autophagy likely served as a mechanism for cell survival, while higher concentrations (15, 20 and 25 μg/mL) of TiO_2_ NPs in the culture media could further influence the cell viability on long term by autophagy. These data are consistent with similar studies that have shown that TiO_2_ NPs are able to disrupt p53 expression and induce autophagy, even under less toxic conditions [27,62]. This defense mechanism saves cells, but after prolonged exposure, these NPs could have harmful potential, which should not be underestimated [63]. Another study conducted in 2016 suggested that the presence of metal oxide NPs in cell slows the degradation of autophagic substrates, because NP aggregates that form at high doses and long-term exposure are difficult to be digested and can overload the cell’s autophagy capacity [64].

Moreover, the cell death observed within this study could be a consequence of the lysosomal degradation induced by the NPs tested. Their ability to generate ROS at the intracellular level has a significant potential to induce lysosomal membrane permeabilization, allowing the release of lysosomal proteases and other hydrolases into the cytosol. The released cathepsins activate the Bid protein, triggering a proteolytic cascade that leads to the activation of caspase-3, and the execution of cell death using the apoptotic pathway, mediated by lysosomal proteases [65,66]. This mechanism of cytotoxicity induced by TiO_2_–Fe(1%)-N (pH~8.5) NPs was confirmed in the present research through cathepsin B immunocytochemical staining (Figure 10B). The quantity of NPs added onto the cells exerted an important modulating action on the protein and gene expression, revealing that there is a threshold concentration of the TiO_2_-based NPs responsible for the shift from a classical pathway of cell death through apoptosis, to a death mechanism related to NP-induced lysosomal disfunction.

## 4. Materials and Methods

### 4.1. Synthesis and Physicochemical Characterization of TiO_2_ NPs

The TiO_2_–Fe(1%)-N co-doped NPs were synthesized hydrothermally, using appropriate amounts of FeCl_3_ × 6H_2_O and TiCl_3_ that were mixed using energetic stirring in ultrapure water; the pH value was adjusted to 8.5 with a solution of 25% NH_4_OH. Afterwards, the resulting co-precipitates were washed repeatedly with ultrapure water and kept at 105 °C until dry. In the next step, the nitrogen doping was achieved by hydrothermal treatment in the presence of urea (200 °C/2 h), followed by powder calcination in air (400 °C/2 h).

The X-ray diffraction (XRD) measurements were performed using a Bruker D8 Advance diffractometer (Bruker, Hamburg, Germany) with Cu Kα radiation (λ = 1.5406 Å). The presence of iron and nitrogen in the TiO_2_ NPs was confirmed through ^57^Fe Mössbauer transmission spectroscopy methods, using a WissEL-ICE Oxford Mössbauer cryomagnetic system (Wissenschaftliche Elektronik GmbH, Starnberg, Germany, and ICE Innovative cryogenic system, Oxford, UK) and X-ray photoelectron spectroscopy (XPS), carried out in an analysis chamber equipped with a 150 mm hemispherical electron energy analyzer (Phoibos, SPECS Gmbh, Berlin, Germany), a dual anode (Mg/Al Kα) X-ray source, and a monochromatized (Al Kα/Ag Lα) X-ray source. The complete procedure was previously described [24].

The morphology of TiO_2_ NPs was evaluated using a transmission electron microscope operating at 200 kV (JEOL JEM—ARM200F, JEOL Inc., Tokyo, Japan). For TEM investigations, the samples were prepared by crushing the TiO_2_–Fe(1%)-N powder in ethanol, followed by ultrasonic dispersion and dropping the particle suspension on lacey carbon grids. More than 100 particles were considered for statistical measurements. Additionally, the surface morphology of the modified TiO_2_ NPs was revealed using a scanning electron microscope (SEM; Quanta 200, FEI, Eindhoven, The Netherlands), equipped with a large field detector (LFD) operating at 15 kV. Before the investigation, the TiO_2_-based NPs were dispersed in pure ethanol, sonicated for 1 h, and then a drop of the obtaining suspension was deposited on a thin glass plate and allowed to dry in UV light.

The photocatalytic efficiency of TiO_2_–Fe(1%)-N NPs in the degradation of methylene blue in the visible light domain (λ > 400 nm) was investigated using a PCC-2 (ULVAC RIKO, Chigasaki, Kanagawa, Japan) photocatalytic checker. For analyzing the photocatalytic behavior of co-doped TiO_2_ NPs, three layered films were prepared, as first described in our previous work [24].

### 4.2. Cell Culture and NPs Exposure

In this experiment, MRC-5 cells, represented by normal human lung fibroblasts (MRC-5 cell line, purchased from ATCC Cat. No. CCL-171) were used. Cell culture was maintained at 37 °C in a humidified atmosphere, with 5% CO_2_, and the growth medium used was complete Eagle’s minimum essential medium (MEM; Gibco/Invitrogen, Carlsbad, CA, USA), containing 2 mM L-glutamine and 4.5 g/L glucose, and supplemented with 10% fetal bovine serum (FBS; Gibco/Invitrogen, Carlsbad, CA, USA). The culture MEM medium was removed every two or three days, and replaced with a fresh medium until the cell culture reached approximately 80% confluence. Afterwards, the cells were detached using a solution with 0.25% (*w*/*v*) Trypsin and 0.53 mM EDTA (Sigma-Aldrich, St. Louis, MO, USA), and subcultured in order to ensure their proper growth and health.

A 5 mg/mL stock suspension of TiO_2_–Fe(1%)-N (pH~8.5) NPs was prepared in phosphate-buffered saline (PBS) and sterilized using an autoclave (120 °C/20 min). The fibroblasts were counted using a hemocytometer with a double counting chamber and a light optical microscope, and then cultured at 2 × 10^4^ cells/cm^2^ density in 25 cm^2^ (for Comet assay, cell viability, flow cytometry, RT-PCR and 8-OHdG analyses) or 75 cm^2^ (for Western blot assays) culture flasks and left overnight to adhere. Next, the fibroblasts were exposed to increasing concentrations (0.001, 0.01, 0.1, 1, 10, 15, 20 and 25 μg/mL) of TiO_2_–Fe(1%)-N (pH~8.5) NPs for 24, 48 and 72 h. In parallel, untreated cells were used for each test and considered as controls At the end of each exposure interval, changes in cell morphology were investigated using phase contrast microscopy (Olympus IX71, Tokyo, Japan).

### 4.3. Cell Viability

For cell viability measurements, the lung fibroblasts were detached, as previously described in Section 2.2, and counted using a Trypan blue exclusion assay. Briefly, 15 μL of MRC-5 cell suspension was added to an equal volume of 0.4% (*w*/*v*) Trypan blue solution, prepared in 0.81% NaCl and 0.06% (*w*/*v*) dibasic potassium phosphate (Sigma-Aldrich, St. Louis, MO, USA). The cell viability was calculated using the following formula:

% viable cells = [1.00 − (Number of blue cells/Number of total cells)] × 100.

### 4.4. Intracellular ROS Level Measurement

The level of intracellular ROS was determined using a fluorescent 2′,7′-dichlorofluorescein diacetate (DCFH-DA, Sigma-Aldrich, St. Louis, MO, USA) compound. The lung cells were washed with PBS solution and then incubated with the dye (37 °C/30 min). Further, the excess dye was removed, and the fibroblasts were resuspended in PBS solution and detached by scraping. The fluorescence relative units were quantified using a fluorimeter (FP-750 Spectrofluorometer, Jasco, Tokyo, Japan) (λexcitation= 488 nm and λemission = 515 nm). The fluorescence intensity obtained was divided by the number of living cells in each sample and, thus, all results were expressed as relative to the control.

### 4.5. Preparing Cell Lysates

The lung fibroblasts were harvested from culture flasks, washed with PBS solution and lysed (30 s × 3 times) on an ice bath with an ultrasonic processor (Hielscher UP50H, Teltow, Germany). The cellular homogenate was centrifuged (3000× *g*/4 °C/10 min) and the supernatants were collected and preserved at −80 °C for the future biochemical assays. The protein concentration of the cellular lysates was measured using the Bradford method that requires Bradford Reagent (Sigma-Aldrich, St. Louis, MO, USA) and a bovine serum albumin (BSA) standard curve.

### 4.6. Western Blot

Western blot analyses were performed in order to assess the expression of proteins involved in different cell death pathways, such as apoptosis (p53, NF-kB, MDM-2, Bax, Bcl-2 and pro-caspase 3) and autophagy (LC3). Therefore, equal quantities (60 μg) of protein from the cell lysates collected for each sample were heated (95 °C/5 min) and separated by 10% SDS-PAGE under reducing conditions at 90 V for 2 h, using a TRIS-glycine buffer (50 mM Tris-HCl (pH 8.3), 50 mM glycine, 0.1% sodium dodecyl sulfate) (Sigma-Aldrich, St. Louis, MO, USA). After electrophoresis, all proteins were transferred to a 0.45 μm polyvinylidene difluoride (PVDF) membrane (Millipore, Billerica, MA, USA) at constant amperage (350 mA) for 95 min in a TRIS-glycine buffer (48 mM Tris-HCl (pH 8.3), 39 mM glycine, 20% methanol) using a wet transfer unit (BIO-RAD, USA). Afterwards, all remaining binding surfaces of the membranes were blocked for 30 min at room temperature, using the blocking buffer solution provided in the Western Breeze Chromogenic kit (Invitrogen, Grand Island, NY, USA) and incubated overnight at 4 °C with appropriate dilutions (1:250) of specific mouse (anti-p53; MDM-2; pro-caspase 3; Bcl-2; Bax; β-actin) (all from Santa Cruz Biotechnologies Inc., Santa Cruz, CA, USA) and rabbit (anti-NF-kB; LC3) (Millipore, Billerica, MA, USA) monoclonal antibodies. Then, PVDF membranes were treated according to the instructions included in the Western blot chromogenic immunodetection kit, using anti-mouse and anti-rabbit secondary antibodies conjugated with alkaline phosphatase and 5-bromo-4-chloro-30-indolyl phosphate/nitroblue tetrazolium (BCIP/NBT) as the chromogenic substrate. The images of Western blot membranes were captured using the GeneSys software (Syngene, Cambridge, UK) and the protein bands were quantified using GelQuantNET software. β-actin was used as a loading control to normalize protein expression, and the values of the protein of interest in NPs-treated cells were expressed as relative to the control for each experiment.

### 4.7. RT-qPCR

The total RNA was extracted from lung fibroblasts using a TRIzol-based method and reverse transcribed into complementary DNA using the iScript Reverse Transcription Supermix for RT-qPCR (BioRad). The Real-Time PCR reactions were carried out on the iCycler iQ Real-Time PCR Detection System (BioRad), using the iQ SYBR Green Supermix Kit (BioRad) and pairs of specific primers designed to amplify sequences from the target genes (Table 4). The analysis was carried out three times for each gene, and the validity of the qPCR method was confirmed by analyzing the melting curves. The relative expression value (2^−ΔCq^) for each gene of interest was obtained by normalizing to the arithmetic mean of the 18S RNA and gapdh reference genes.

### 4.8. Comet Assay

After cells’ exposure to 0.01 and 25 µg/mL of TiO_2_–Fe(1%)-N NPs for 24, 48 and 72 h, DNA damage was analyzed using a commercially available single cell gel electrophoresis kit (Cell Biolabs, INC, San Diego, CA, USA). MRC-5 cells were collected and resuspended and diluted in ice-cold PBS solution at 1 × 10^5^ cells/mL density. An amount of 8 µL of each cell suspension was mixed with 80 µL of low melting agarose, but only 75 µL were spread on a comet slide. The comet slides were maintained horizontally in the dark (4 °C/15 min) and then transferred to pre-chilled lysis solution (4 °C/1 h) provided in the kit. Further, the lysis solution was discarded, and the comet slides were submerged in the alkaline solution in the dark (4 °C/30 min). After that, the comet slides were washed twice for 5 min each with pre-chilled deionized water, placed in a horizontal gel tank and electrophoresed at a low voltage (300 mA, 25 V) for 30 min. After electrophoresis, the comet slides were immersed in 70% ethanol for 5 min, and allowed to dry, before staining them with a Vista Green DNA dye for 15 min. The comets were visualized through fluorescence microscopy (Olympus IX 71, Tokyo, Japan), using the fluorescein isothiocyanate (FITC) filter. The results were analyzed and processed using the Comet Assay IV software. The percentage of DNA in the tail (%T) was chosen as a mark of DNA damage. There are many tests that interfere with the nanoparticles’ properties, but in the case of the Comet assay, we did not observe any interferences, which is in accordance with other previous papers that used this test [67,68].

### 4.9. Evaluation of Oxidative DNA Damage

At the end of each exposure period, DNA extraction and purification from the collected cells was performed, followed by DNA digestion with P1 nuclease and alkaline phosphatase. Next, the samples were processed using the “8-hydroxy 2-deoxyguanosine (8-OHdG) ELISA” kit (Abcam cat. No. Ab201734), according to the manufacturer’s instructions.

### 4.10. Assessment of TiO_2_-NPs Treated Fibroblasts Distribution over the Cell Cycle by Flow Cytometry

MRC-5 cells exposed to 0.01 and 25 µg/mL of TiO_2_–Fe(1%)-N NPs for 24, 48 and 72 h were harvested, washed with PBS and centrifuged at 1500 rpm for 5 min. A positive control consisting of MRC-5 cells exposed to 25 µg/mL of dimethyl sulfoxide was also used. The cells were fixed with 500 μL of 70% ice-cold ethanol (4 °C/1 h). After two successive washes with PBS solution, the cells were incubated with 0.5 mg/mL RNase A (37 °C/30 min) and then stained with propidium iodide (50 μg/mL) solution for 15 min in the dark. The red fluorescence of 10,000 events in propidium iodide-stained cells was acquired in a FL4 Log channel, through a 675 nm band-pass filter using a BD Accuri C6 flow cytometer (BD Biosciences, San Jose, CA, USA). The data were analyzed, excluding the cell debris (characterized by a low FSC/SSC), using the software provided with the BD Accuri C6 flow cytometer.

### 4.11. Lysosomes Distribution Analysis

Lysosomes were stained with 100 nM LysoTracker Green DND-26 (Molecular Probes, Invitrogen) (37 °C/5% CO_2_/30 min), and nuclei were counterstained with 2 μg/mL Hoechst 33342 (Molecular Probes, Invitrogen) after a 10 min incubation at room temperature. Lysosomes’ distribution was investigated using fluorescence microscopy (Olympus IX71, Tokyo, Japan).

### 4.12. Cathepsin B Immunocytochemistry

MRC-5 cells were seeded at 2 × 10^4^ cells/cm^2^ density on coverslips and allowed to firmly adhere overnight. Afterwards, cells were exposed to 0.01 and 25 µg/mL of TiO_2_–Fe(1%)-N NPs for 24, 48 and 72 h. At the end of each time interval, the cells were washed with PBS, fixed with 4% paraformaldehyde for 20 min at room temperature, permeabilized with 0.1% Triton X-100—2% BSA for 30 min, and incubated at 4 °C overnight with Alexa Fluor 594 conjugated fluorescent primary anti-cathepsin B antibody (Santa Cruz). Nuclei were counterstained with 2 μg/mL 4′,6-diamidino-2-phenylindole (DAPI) (Molecular Probes, Life Technologies, Carlsbad, CA, USA). Cells were analyzed for vesicular versus diffuse staining of lysosomal cathepsin B using a fluorescence microscope (Nikon Eclipse E200, Tokyo, Japan) with a 60× objective.

### 4.13. Statistical Analysis

Data were expressed as the mean value ± standard deviation (SD) of the three independent experiments (*n* = 3). Statistical differences between the samples and the control were evaluated by applying Student’s *t*-test, using the GraphPad Prism software (version 5; GraphPad Software, Inc., La Jolla, CA, USA), and a value of *p* < 0.05 was expressed as being statistically significant.

## 5. Conclusions

The present study showed that the exposure of lung fibroblasts to concentrations higher than 10 ng/mL of TiO_2_–Fe(1%)-N (pH~8.5) NPs for 72 h induced oxidative stress, disruption of the cell cycle phases, DNA damage, cell death by autophagy and apoptosis. In addition, our research elucidated a mechanism for triggering intrinsic apoptosis on a p53-independent and ROS-dependent pathway, mediated by lysosomal proteases. In the context of the ongoing worldwide attempts to allow TiO_2_ NPs to reach their full potential as a photocatalyst, the results of our work contribute to the better understanding of the mechanisms of interaction of functionalized TiO_2_ NPs with in vitro biological systems. This knowledge is of great importance in obtaining new biocompatible, and more efficient, nano-photocatalysts.

## Figures and Tables

**Table 1:** Lattice parameters, crystallite size, phase assignment and relative abundance of the TiO_2_–Fe(1%)-N (pH~8.5) sample.

Sample	Lattice Parameters (Å)			Crystallite Size (nm)	Phase Assignment/ Abundance (wt%)
	*A*	*b*	*c*		
TiO_2_–Fe(1%)-N (pH~8.5)	3.7912	-	9.4909	12.3	Anatase (85.3)
	9.1429	5.4215	5.2450	8.5	Brookite (14.7)
Errors	±0.0005	±0.0005	±0.0005	±1.5	±1.4

**Table 2:** The binding energy values extracted from the deconvolutions of the X-ray photoelectron spectroscopy (XPS) spectra.

Sample	Ti 2p_3/2_	Fe 2p_3/2_	N 1s
	Binding Energy (eV)		
TiO_2_–Fe(1%)-N (pH~8.5)	456.88	710.55	398.60
	458.62		400.50

**Table 3:** Activation ratio of caspase-3 in MRC-5 fibroblasts exposed to TiO_2_ NPs. The protein expression was normalized to β-actin. Results are expressed as the mean ± standard deviation (SD) (*n* = 3).

Time (h)	Control	Cleaved Caspase-3/Procaspase-3 Ratio							
		TiO_2_–Fe(1%)-N (pH~8.5) NPs (μg/mL)							
		0.001	0.01	0.1	1	10	15	20	25
24	0.36 ± 0.12	0.36 ± 0.06	0.35 ± 0.02	0.34 ± 0.09	0.34 ± 0.02	0.35 ± 0.06	0.34 ± 0.01	0.34 ± 0.27	0.37 ± 0.02
48	0.29 ± 0.04	0.27 ± 0.01	0.26 ± 0.03	0.26 ± 0.02	0.26 ± 0.09	0.27 ± 0.04	0.27 ± 0.03	0.27 ± 0.06	0.26 ± 0.1
72	0.036 ± 0.001	0.044 ± 0.001	0.043 ± 0.001	0.05 ± 0.001	0.09 ± 0.001 *	0.13 ± 0.001 **	0.3 ± 0.002 **	0.72 ± 0.015 *	5.23 ± 0.058 *

Notes: * *p* < 0.05 and ** *p* < 0.01 compared to control.

**Table 4:** Sequences of forward and reverse primers for qPCR analysis.

Gene	Primer Name	Primer Sequence
*BAX*	BAX-F	5′-GGCTGGACATTGGACTTC-3′
	BAX-R	5′-GTGAGGAGGCTTGAGGAG-3′
*TP53*	TP53-F	5′-ACCTATGGAAACTACTTCCTGAAA-3′
	TP53-R	5′-CTGGCATTCTGGGAGCTTCA-3′
*NFkB*	NFkB-F	5′-GCAGCACTACTTCTTGACCACC-3′
	NFkB-R	5′-TCTGCTCCTGAGCATTGACGTC-3′
*18S RNA*	18S-F	5′-GCTTAATTTGACTCAACACGGGA-3′
	18S-R	5′-AGCTATCAATCTGTCAATCCTGTC-3′
*gapdh*	gapdh-F	5′-TGGTCTCCTCTGACTTCAAC-3′
	gapdh-R	5′-GTGAGGGTCTCTCTCTTCCT-3′

Notes: BAX is Bcl-2-associated X protein; TP53 is tumor protein p53; NFkB is nuclear factor kappa B; 18S RNA is 18S ribosomal RNA; gapdh is glyceraldehyde-3-phosphate dehydrogenase.

## Data Availability

Data are available on request from the corresponding author.
